# Atmospheric Ionic Deposition in Tropical Sites of Central Sulawesi Determined by Ion Exchange Resin Collectors and Bulk Water Collector

**DOI:** 10.1007/s11270-012-1211-8

**Published:** 2012-05-26

**Authors:** S. Köhler, H. F. Jungkunst, C. Gutzler, R. Herrera, G. Gerold

**Affiliations:** 1 Landscape Ecology, Institute of Geography, University of Göttingen, Goldschmidtstr. 5, 37077 Göttingen, Germany; 2 Landscape Ecology and Land Evaluation, University of Rostock, Justus-von-Liebig-Weg 6, 18051 Rostock, Germany; 3 Geoecology/Physical Geography, Institute for Environmental Science, University of Koblenz-Landau, Fortstraße 7, 76829 Landau, Germany; 4 Department of Hydraulic Engineering and Environmental Sciences, Universidad Politécnica de Valencia, Valencia, 46022 Spain

**Keywords:** Bulk deposition, Central Sulawesi, Passive collector, Nitrate deposition, Phosphorus deposition

## Abstract

In the light of global change, the necessity to monitor atmospheric depositions that have relevant effects on ecosystems is ever increasing particularly for tropical sites. For this study, atmospheric ionic depositions were measured on tropical Central Sulawesi at remote sites with both a conventional bulk water collector system (BWS collector) and with a passive ion exchange resin collector system (IER collector). The principle of IER collector to fix all ionic depositions, i.e. anions and cations, has certain advantages referring to (1) post-deposition transformation processes, (2) low ionic concentrations and (3) low rainfall and associated particulate inputs, e.g. dust or sand. The ionic concentrations to be measured for BWS collectors may easily fall below detection limits under low deposition conditions which are common for tropical sites of low land use intensity. Additionally, BWS collections are not as independent from the amount of rain fallen as are IER collections. For this study, the significant differences between both collectors found for nearly all measured elements were partly correlated to the rainfall pattern, i.e. for calcium, magnesium, potassium and sodium. However, the significant differences were, in most cases, not highly relevant. More relevant differences between the systems were found for aluminium and nitrate (434–484 %). Almost five times higher values for nitrate clarified the advantage of the IER system particularly for low deposition rate which is one particularity of atmospheric ionic deposition in tropical sites of extensive land use. The monthly resolution of the IER data offers new insights into the temporal distribution of annual ionic depositions. Here, it did not follow the tropical rain pattern of a drier season within generally wet conditions.

## Introduction

Human impact on atmospheric depositions is high (Pye et al. 2009; Fan et al. 2009; Aas et al. 2007) and particularly depositions of nitrogen (N), sulphur (S), phosphorus (P) and potassium (K) have considerable influence on ecosystems (Cinderby et al. 1998; Adams 2003; Rockström et al. 2009). N and P cycles are among the most heavily influenced earth system processes by human (Rockström et al. 2009; Phoenix et al. 2006). Even for remote tropical areas, a substantial increase of N deposition is assumed and its potential impacts are being investigated (Corre et al. 2007; Phoenix et al. 2006). A growing number of studies in the northern hemisphere (Bytnerowicz et al. 2002; Fenn et al. 2000; Fenn and Poth 2004; Gassmann 2004) have shown increasing atmospheric depositions related to human activities and associated environmental impacts (Fan et al. 2009; Aas et al. 2007; Curtis et al. 2009; Phoenix et al. 2006). For example, an excess of N leads to a multitude of undesirable reactions of the affected ecosystems, which can be: (a) acidification (Adams 2003), (b) contamination of ground and surface water (Curtis et al. 2009) and (c) increasing greenhouse gas emissions, for example N_2_O (Veldkamp et al. 2008). Another major impact of atmospheric N deposition is the associated loss of richness of species (Phoenix et al. 2006). The latter is extraordinarily important for the tropics which are general biodiversity hot spots. Some studies performed in tropical forests, far from urban areas, have reported alarming increases in deposition rates (Aas et al. 2007; Fan et al. 2009). Thus, monitoring atmospheric ionic deposition across tropical sites is needed and suitable methods have to be explicitly simple and robust to minimise the effects of high temperatures and air humidity. Furthermore, simple economic methods have multiple advantages particularly for poor and remote tropical regions.

In the tropics, it is even more difficult than for most other areas to perform precise atmospheric deposition monitoring for a larger area which usually includes very remote sites. Additional to the typically extremely poor accessibility and lack of electricity, which applies for many areas outside the tropics as well, high temperatures and high humidity provoke fast biochemical reactions in the collected samples before they can be analysed. The most relevant problem of “conventional” bulk water collectors (BWS) is concentrations that are too low to be analysed precisely by most common devices. Frequently, samples for nitrate (NO_3_–N) and P show concentrations that are below the detection limits for most laboratories, particularly in the tropics. This holds the risk of great uncertainties particularly for low input site that are still near pristine conditions. These sites are especially important as reference sites and monitoring sites to document the expected changes in depositions in the tropics.

A possibility to overcome this general problem of concentrations that are frequently below detection limits is to use passive collectors based on ion exchange resins (IER) which have been tested successfully before particularly for anions (Crabtree and Trudgill 1981; Simkin et al. 2004; Fenn et al. 2002; Fenn and Poth 2004; Templer and Wethers 2011). Our own preliminary tests in the laboratory show general agreement with the reported results and confirm the suitability of this method for cations as well. In theory, ions (cations and anions) from rainwater are adsorbed on the surface of the resin during percolation through simple constructed ion exchange resin collectors (IER collectors). Consequently, concentrations rise with time and become more likely detectable when resins are extracted at the laboratory after a longer period. Temporal resolution might be reduced, but on the other hand, it is highly likely that input values would be less uncertain. However, Fenn and Poth (2004) have shown that particularly for the anion NH_4_^+^, background levels from blank IER collectors can be substantial and have to be considered. Otherwise, artificially high values are monitored. Furthermore, some extraction solutions like sodium citrate do not exchange ions successfully (Simkin et al. 2004). Another limitation of the IER collectors is that the used anion or cation for extraction of course cannot be used for environmental monitoring.

The designs of passive collectors vary and so does the choice of exchange resins of different capacity. Our main goal was to construct an ideal passive collector for the tropics to be used at a monthly resolution scale. The objective was to compare the amount of the total deposition per period collected by the “classic” bulk water sampling method with the values derived from the IER collectors. We expected that by extracting the resin with a small volume of extracting medium, the concentration in the extract would be well above the detection limit. Consequently, our hypothesis was that adapted passive collectors could provide more reliable results for low deposition rates than the “classic bulk water collectors” for aluminium (Al), calcium (Ca), K, iron (Fe), magnesium (Mg), sodium (Na), P and NO_3_–N. A simple, economic design for easy operation in remote field locations would also help in enhancing possibilities to study deposition rates in tropical countries. The objective was to improve our knowledge on atmospheric ionic deposition for Central Sulawesi. The focus was on the particularity of atmospheric ionic deposition in tropical sites with low land use intensity at a monthly resolution. Our goal was to improve the temporal interpretation of bulk depositions. Do bulk ionic depositions mainly follow the rain pattern, i.e. are low depositions typical for the dry season?

## Materials and Methods

### Study Sites

**Fig. 1:**
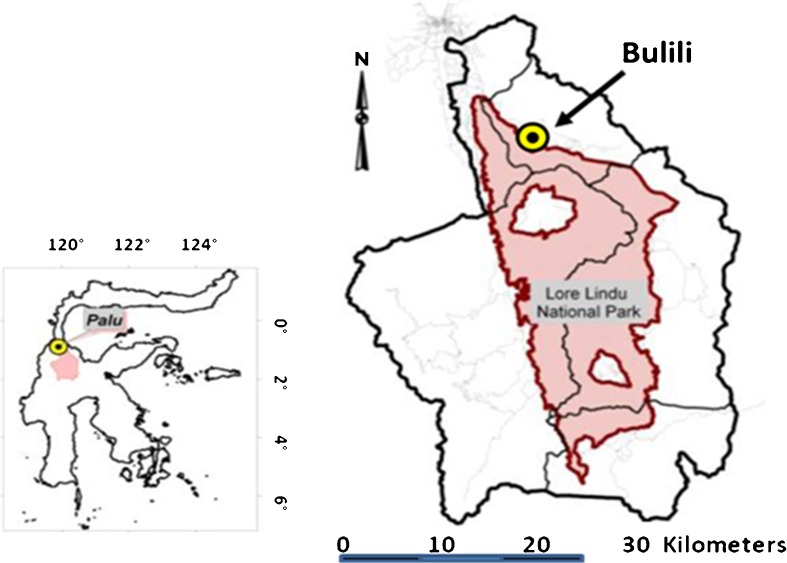
Study site near the Lore Lindu National Park in Central Sulawesi, Indonesia

The comparisons of the different measuring systems were done at three sites close to the village of Bulili (1°12′ S, 120°05′ E) near the Lore Lindu National Park, Sulawesi, Indonesia. The sites were located at different altitudes between 632 (site A), 817 (site B) and 926 m asl (site C), along a mountain transect. The lower sites were near cacao plantations, while the upper site was located near the rainforest. Total average annual rainfall of the research area is around 2,500 mm a^−1^. The mean annual temperature in the study area is 24.5°C, with a mean relative high humidity of 85 %. In a test of evaporation at the study site (Fig. 1), evaporation under 0.5 mm per week was measured (Gutzler 2011).

### Sampler Design

At each site, three pairs of conventional bulk water collectors (BWS collectors) and ion exchange resin collectors (IER collectors) were installed in an area of 2 m in diameter. Both systems are based on the same collecting principle for bulk deposition from precipitation. The bulk deposition sampler consists of a funnel (polyethylene) of 283.53 cm² at the top connected to a PVC tube with a diameter of 8 mm. For both systems, the top of the funnel was levelled and fixed to 1 m above the ground. The construction of the samplers is easy and performed with materials available at local markets.

**Fig. 2:**
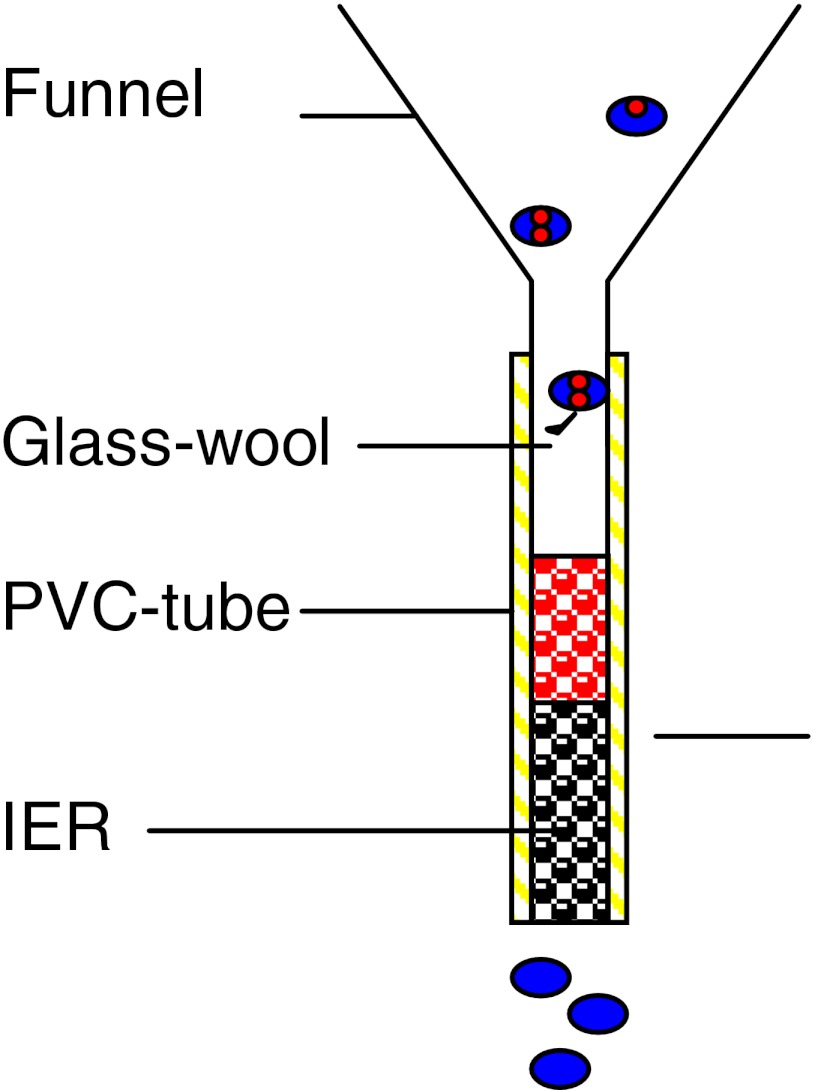
Scheme of an IER collector

For the BWS collector, a 75-cm-long PVC tube was inserted into a 5-l can for collecting rainwater. The deposition rate was calculated from the volume and concentration of elements in the rainwater in relation to the funnel area and the sampling interval. Because of tropical high mean temperature, relevant evaporation rates were expected but Gutzler (2011) found no relevant evaporation for the BWS collectors. For the IER collector, the same funnel (polyethylene) and tube types were used. The 50-cm tube of the IER collector was closed with a net at the end to retain the resin inside and filled with 50 g ion exchange resin amberlite MB 20 (Fig. 2). Amberlite MB 20 is an anion and cation mixed ion exchange resin with a capacity of about 0.5 mmol charge per gram. The ion content of the rainwater is adsorbed at the surface of the ion exchange resin. These ions can be remobilised later by extracting the ions from the exchange resin at the laboratory using acids or NaCl solutions. The resin is highly stable even under very low pH or high salt concentrations. To prevent leaves or insects from entering the column, the resin package was covered with glass wool (Fig. 2). For sampling, the IER collector, whole tube filled with ion exchange resin, was replaced at the field site and brought to the laboratory for preparation and analyses. IER collectors were tested in the laboratory before the field study.

### Field Sampling

The sampling interval for the BWS collector was twice a week. The total amount of rainwater, collected in the 5-l can, was measured with a graduated cylinder. From each BWS collector, a 100-ml subsample for analyses was taken to the laboratory. To sample the IER collector, the whole tube with the ion exchange resin was brought to the laboratory.

Site B was installed first and sampled twice a month from 26 January 2007 until 30 May 2007. The aim of the higher sampling rate was to test the capacity of ion exchange resin in the field, with deposition rates similar to those given by Niklas (2006). The following samplings until 31 December 2008 were done once per month. For identifying possible spatial variations of yearly results, additional sites A and C were sampled monthly starting on 29 February 2008 until 31 December 2008.

From the beginning, the elements Ca, K, Mg and Na were analysed. Starting with the 1 March 2007 sampling, NO_3_–N, and from 2 August 2007, P and the microelements Al, Fe and Mn were also measured.

### Sample Preparation and Routine Analyses

The water samples and extract were measured using an inductively coupled plasma–optical emission spectrometry (ICP-OES) 2000 DV (Perkin Elmer). NO_3_–N was analysed using a continuous flow analysis (CFA) System AA3 Autoanalyser (Bran & Luebbe). For sample preparation, the water samples had to be washed and filtered with 0.45-μm filter from Sartorius and directly analysed at the ICP-OES and CFA System. The ion exchange resin subsamples were weighed and homogenised. Approximately a 10-g subsample was taken and extracted twice with 50 ml sulphuric acid (2 M) to determine cations and P. To measure NO_3_–N, the extraction of another 10 g ion exchange resin was done with sodium chloride (1 M) solution. In order to correct the calculations of the deposition rate, a blank and an extraction factor were measured under the same conditions in the lab with unused ion exchange resin. Therefore, under controlled conditions, unused ion exchange resin was percolated with different concentrated solutions. The percolated water was analysed for unfixed elements passing through to calculate a correction recovery factor for each element separately. For the blank, a subsample of unused ion exchange resin was extracted and analysed. Both factors were used to calculate the results (Table 1).

**Table 1:** Blanks of ion exchange resins and extraction factor for different elements

	Al	Ca	Fe	K	Mg	Mn	Na	NO_3_–N	P
Blank IER (mg g^−1^)	0.008	0.0039	0.0020	0.0013	0.0051	0.0001	0.0043	0.0009	0.0011
Factor	1.558	2.121	1.533	1.089	1.602	1.247	1.091	0.952	1

## Results

The deposition rates determined with both systems for the ions Ca, Mg, K and Na at site B exhibited nearly the same cumulative values for 19 months (Table 2). The relative comparison revealed 3, 7 and 14 % higher values for the IER collectors compared to the BWS collectors for Mg, Ca and Na, respectively. For K, the IER collectors sampled 35 % lower values than the BWS collector. NO_3_–N was measured simultaneously by both systems for 18 months and the values for IER collectors showed 434 % higher deposition rates than the BWS collectors. All differences were statistically significant (Table 2).

**Table 2:** The cumulative deposition rates for IER collectors and conventional rain collectors at Bulili, field site B, Sulawesi, Indonesia

	Time period month	BWS collector (mg m^−2^)	Standard deviation	IER collector (mg m^−2^)	Standard deviation	IER/BWS (%)
Part A						
Ca	19	1,725	86	1,851	59	107
K	19	1,681	94	1,100	41	65
Mg	19	329	16	339	10	103
Na	19	2,168	118	2,464	111	114
NO_3_–N	149	49	12	214	19	434
Part B						
Al	12	25	2	121	7	494
Fe	12	66	4	87	6	132
Mn	12	16	2	13	1	80
P	12	110	9	127	7	116

Different measuring systems with a significant difference at *p* = 0.05

Furthermore, Fe, Mn, P and Al were measured for 1 year beginning in August 2007 (Table 2). For P, the difference between both systems was 17 %, which is similar to Ca, Mg and Na. A comparable difference of 19 % was analysed for Mn, but as for K, the IER collectors collected less than the BWS collector. Slightly higher differences were found for Fe. Again, the IER collectors measured higher deposition of 34 %. More relevant and significant differences of 384 % higher inputs were registered with the IER collectors for Al.

At the experimental sites A and C, significant differences between the systems were found for Fe, Mn and NO_3_–N. Annual depositions rates are shown in Table 3.

**Table 3:** Yearly deposition in kilograms per hectare per annum for different elements at three experiment sites

	Al	Ca	K	Mg (kg ha^−1^ a^−1^)	Mn	Na	NO_3_–N	P
Site A	1.6	15.5	5.7	1.8	0.1	23.9	2.7	1.3
Site B	1.1	10.4	7.5	3.2	0.1	19.6	1.4	1.2
Site C	1.0	14.9	10.6	2.6	0.2	20.0	2.0	1.2

### Seasonal Variations in Precipitation and Deposition Rates

**Fig. 3:**
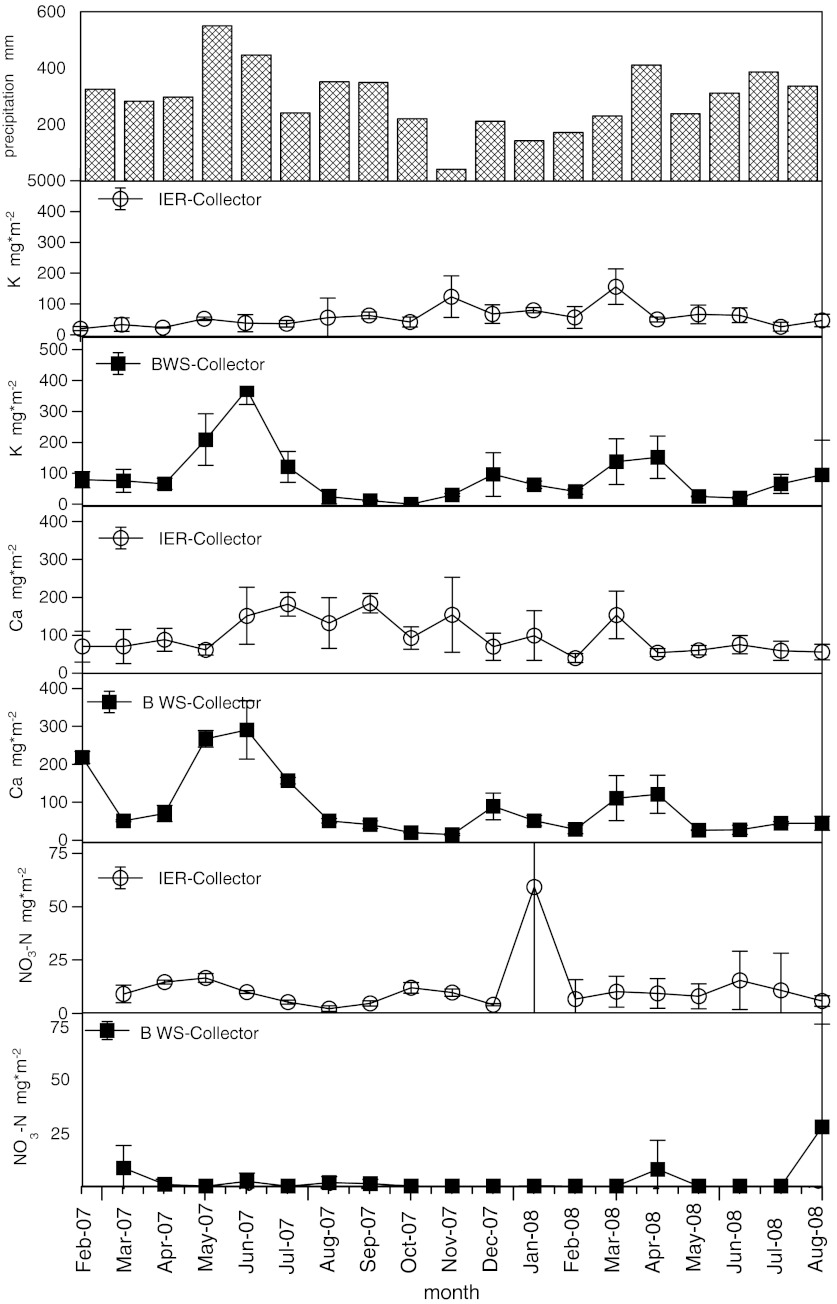
**a**–**e** Monthly precipitation is given in (**a**). **b**–**f** Deposition rates over the experimental time at the site open area for the elements K, Ca and NO_3_–N collected with bulk water collector or ion exchange resin collector systems

In order to study the seasonal variations in deposition rates as related to rainfall pattern, three ions were picked as examples for: (a) lower (K), (b) higher (Ca) and (c) extremely higher (NO_3_–N) values measured with the IER collector system as compared to the BWS collector system (Fig. 3a–f). During the experimental time, the highest monthly precipitation of 550 mm was registered for May 2007. November 2007 was the period with the lowest precipitation of 41 mm during the whole experiment (Fig. 3a). The monthly deposition rates of K measured with the IER collector were rather steady. Only during November 2007 and March 2008 that higher depositions with a wider range were registered (Fig. 3b). The deposition rate measured with BWS collectors was, in comparison, more event-driven showing thus high deposition rates corresponding to high precipitation rates. Examples are the months of May till June 2007 as well as March and April 2008 and during the dry periods of November and December 2007 (Fig. 3c). Similar patterns were found for Ca. Rather steady depositions rates were registered by the IER collectors, whereas somewhat lower deposition rates (55 to 87 mg m^−2^ month^−1^) were measured for February until May 2007 (March 2008 was excluded with 153 mg m^−2^ month^−1^) and for February until August 2008 compared to June 2007 until January 2008 which was a period of higher Ca deposition rates (92 to 184 mg m^−2^ month^−1^) (Fig. 3d). The Ca deposition rates measured by the BWS collector followed the rain events of May and June 2007 (267 to 290 mg m^−2^ month^−1^) as well as March and April 2008 (111 to 121 mg m^−2^ month^−1^) with higher deposition rates (Fig. 3e).

The IER collector clearly revealed more steady deposition rates than the BWS collector particularly for NO_3_–N. NO_3_–N deposition rates were solely registered in 7 out of 18 months with the BWS collector. The high amount in August 2008 was found in one of three replications (0, 0 and 82 mg m^−2^) for NO_3_–N, and for the same sampler, the K deposition (27, 33 and 225 mg m^−2^) was extremely high. For this event, a contamination, e.g. by birds, cannot be ruled out. Furthermore NO_3_–N depositions were regularly measured only in one of three replications of the BWS collectors. In contrast, the extreme value of 135 mg m^−2^ found in one IER collector in January 2008 was not related with high K depositions. The difference between the IER collector and the BWS collector for the monthly deposition of K and Ca follows the distribution of precipitation. Higher amounts collected by the BWS collector are usually measured when monthly precipitation was over 220 mm. The same phenomenon was found for the other elements except for Na.

**Fig. 4:**
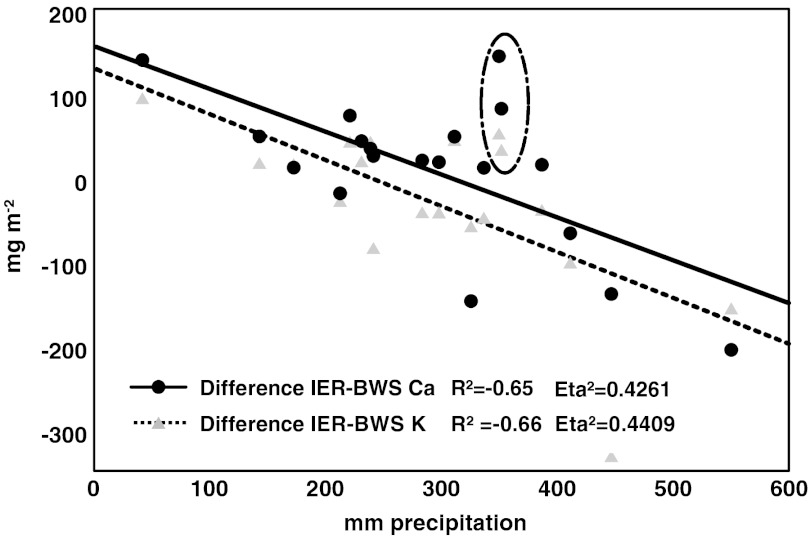
Correlation between the differences of the deposition rate of bulk water collector and IER collector for Ca and K at site B

Differences between IER collector and BWS collector for Ca and K correlate significantly with precipitation (Fig. 4). Exceptions were measured in August and September 2007 (points within the ellipse). Since both elements synchronously showed the irregularity, another driver for deposition could be occurring. Without these two points, the relationship between the difference between IER and BWS showed higher significant correlations for Ca (*R* = −0.81) and for K (*R* = −0.75) with precipitation. No correlation was found for the differences of NO_3_–N and precipitation.

### The Case of Low Deposition

The concentration of ions, extracted from the samples of the IER collector, showed clearly higher concentrations than the water samples from the BWS collector (Table 4). For all elements, 95 % of the IER samples were higher than the lowest standard, Mn was excluded which showed 83 % from all samples under 0.1 mg l^−1^. So, the concentration in most samples from IER collectors falls indisputably within easily detectable ranges of concentrations easily measured in modestly equipped laboratories. For the BWS collector, more than 85–97 % of all samples were lower than 0.1 mg l^−1^. Only for Na, Ca and K most samples were in a range above 0.1 mg l^−1^ (Table 4).

**Table 4:** Percentage of bulk water and IER extract samples below the threshold concentration of 0.1 mg l^−1^

	Al	Ca	Fe	K	Mg (%)	MN	Na	NO_3_–N	P
BWS collector	92.3	13.1	92.0	15.1	84.2	92.4	9.0	97.3	85.3
IER collector	7.0	0.5	4.5	4.2	1.0	83.9	4.0	4.5	1.3

Bulk water samples (total): *n* (Al, Fe, Mn and P) = 570, *n* (Ca, K, Mg and Na) = 680, *n* (NO_3_–N) = 582 IER samples (total): *n* = 380, *n* (NO_3_–N) = 354

## Discussion

The results of the BWS collectors, the method applied by most other authors, generally showed rather low deposition rates. Other studies reporting annual bulk depositions for the tropics were predominately in the range of our IER collector results (Table 3). Mayer et al. (2000), Veneklaas (1990) and Boy et al. (2008) reported, for Latin America, similar ranges for Al (0.12–5.2 kg ha^−1^ a^−1^), Ca (13–76 kg ha^−1^ a^−1^), Na (11–31 kg ha^−1^ a^−1^), Mg (2.2–3.2 kg ha^−1^ a^−1^) and K (6.9–8.3 kg ha^−1^ a^−1^) as we found for Central Sulawesi but with the IER collectors. The deposition rate for P was a little higher than those reported in other studies (0.48–0.72 kg ha^−1^ a^−1^), but Boy et al. (2008) found a similar range of 0.64–1.17 kg ha^−1^ a^−1^. No comparable results were found for Mn deposition and our results were three times higher than those reported for the mountain forest Cordillera Real, Ecuador (Boy et al. 2008) and ten times higher than those reported for the native agri- and silvicultural ecosystems of the Brazilian Cerrado (Lilienfine and Wilke 2004). The NO_3_–N depositions measured with the IER collectors were in the range of low depositions rates (1.8–3.4 kg ha^−1^ a^−1^) found in natural rainforests of Ecuador, Costa Rica and Sulawesi (Eklund et al. 1997; Boy et al. 2008; Dechert et al. 2005). These rates were low in comparison to other NO_3_–N deposition rates measured in African secondary lowland forest (8.1–45 kg ha^−1^ a^−1^) (Lacaux et al. 1992; Muoghalu 2003) or for SE Brazil (6.6–10.7 kg ha^−1^ a^−1^) (Mayer et al. 2000).

Both collector systems showed values that were mostly similar for the total measurement period of 19 months. Nevertheless, the rather small differences were significant except for P, Fe and Mn (Table 2). These small differences mainly remained at the annual perspective, but the collector system that was showing higher values changed in few cases. For example, the IER collector deposition rate was 22 % higher for K measured from August 2007 until July 2008, whereas for the whole period, the deposition rate derived from the BWS collector was 35 % higher (Table 2). Precipitation of Central Sulawesi is characterised by one dryer period per year. During the 19-month measurements, only one dryer period was included. Consequently, the wetter periods were overrepresented which may explain the above-described phenomena of changing differences between the systems. The differences between the two collector systems were most likely related to the different principle of deposition calculations. For the conventional bulk water collector, the concentrations are multiplied by the amount of precipitation. Therefore, deposition rates measured with conventional bulk water collectors were not independent from precipitation rates and wetter periods are unavoidable associated with relative higher deposition rates. This was not the case for IER collectors as virtually all ions are fixed independent of concentration variations of the different precipitation events (Fig. 3). When measuring deposition rates with IER collectors, it is even unnecessary to know the absolute amount of precipitation. Consequently, the differences found between the two systems can be an artefact of calculation. This is indicated by the correlation analyses between the differences of the collector systems to the precipitation in Fig. 4.

These uncertainties are high for BWS if concentrations were close to or below detection limits (Table 4). A good example for that was given by the low NO_3_–N deposition rates detected by this study for Central Sulawesi (Fig. 3). This explained the larger differences between the systems from 35 % up to 484 % found for low depositions of Al, Fe, Mn and NO_3_–N which were mainly lower than 1 kg ha^−1^ a^−1^.

For ions with high to medium deposition rates (>1 kg ha^−1^ a^−1^), namely Ca, Mg, Na and P, differences between the collector systems were less than 20 % (Table 2). Basically, the results of Fenn and Poth (2004), Simkin et al. (2004) and Krupa and Legge (2000) that passive collectors tend to collect slightly higher amounts were confirmed for cations as well.

For a monthly resolution, more relevant and significant differences were found. Particularly, significant differences were found during times of high precipitation (e.g. May 2007) for ions like Ca, K and Mg (Fig. 3). But also, during low precipitation (e.g. November 2007), for ions like Al and Fe, significant differences were found at the monthly resolution. Therefore, different interpretations of temporal dynamics of ionic atmospheric depositions are possible for shorter term analysis depending on the collector system that was applied. It is somewhat striking that deposition values derived from the IER systems hardly show any seasonality as could be expected by precipitation seasonality (dryer and wetter season of Sulawesi). The results of the IER appear more reliable as problems associated with low concentrations were not given.

## Conclusions

The advantage of the classic bulk water collector is the possibility of higher time resolutions and the comparison to more measurements from other researchers. The advantages of the IER system are: (a) ions are fixed, therefore low concentration at or below detection limits can be avoided and (b) biochemical transformation processes in the field during measuring intervals are less likely. The IER system is easy to install and requires little attention once in the field. Collecting the samples for analyses takes a very short time. Consequently, it becomes easy to train local people to perform the sampling routine exchanging the IER collectors. Sampling atmospheric deposition data from remote and large areas is possible with IER systems and allows complex overviews of temporal and potentially spatial variations of deposition inputs. Our knowledge on the atmospheric ionic composition of depositions in rural tropical sites mostly would improve if more such sites were measured with the IER methods. That is assuming the values derived with the IER method are generally more precise mainly due to the advantage of capturing low deposition rates typical for remote tropical sites. For nearly pristine tropical sites, it becomes crucially important to be able to measure low inputs to be used as reference to monitor future changes particularly for nitrate. This is more likely achieved with IER than with BWS.
